# Inhibition of *Staphylococcus pseudintermedius* Efflux Pumps by Using *Staphylococcus aureus* NorA Efflux Pump Inhibitors

**DOI:** 10.3390/antibiotics12050806

**Published:** 2023-04-24

**Authors:** Elisa Rampacci, Tommaso Felicetti, Giada Cernicchi, Valentina Stefanetti, Stefano Sabatini, Fabrizio Passamonti

**Affiliations:** 1 Department of Veterinary Medicine, University of Perugia, Via San Costanzo 4, 06126 Perugia, Italy; 2 Department of Pharmaceutical Sciences, Via Del Liceo 1, 06123 Perugia, Italy

**Keywords:** antibiotic resistance breakers, antimicrobial resistance, efflux pump inhibitors, NorA, *Staphylococcus pseudintermedius*, *Staphylococcus aureus*, new antibiotics

## Abstract

One promising approach in treating antibiotic-resistant bacteria is to “break” resistances connected with antibacterial efflux by co-administering efflux pump inhibitors (EPIs) with antibiotics. Here, ten compounds, previously optimized to restore the susceptibility to ciprofloxacin (CIP) of *norA*-overexpressing *Staphylococcus aureus*, were evaluated for their ability to inhibit *norA*-mediated efflux in *Staphylococcus pseudintermedius* and synergize with CIP, ethidium bromide (EtBr), gentamycin (GEN), and chlorhexidine digluconate (CHX). We focused efforts on *S. pseudintermedius* as a pathogenic bacterium of concern within veterinary and human medicine. By combining data from checkerboard assays and EtBr efflux inhibition experiments, the hits 2-arylquinoline **1**, dihydropyridine **6,** and 2-phenyl-4-carboxy-quinoline **8** were considered the best EPIs for *S. pseudintermedius*. Overall, most of the compounds, except for 2-arylquinoline compound **2**, were able to fully restore the susceptibility of *S. pseudintermedius* to CIP and synergize with GEN as well, while the synergistic effect with CHX was less significant and often did not show a dose-dependent effect. These are valuable data for medicinal chemistry optimization of EPIs for *S. pseudintermedius* and lay the foundation for further studies on successful EPIs to treat staphylococcal infections.

## 1. Introduction

The rise of bacterial pathogens resistant to antibacterial agents poses a major threat to human and animal health worldwide. The ever-increasing demand for novel antimicrobial strategies, in contrast to the current level of investments, has led to the development of several early phase-hit compounds [1]. Among these, efflux pump inhibitors (EPIs) are antimicrobial resistance breakers targeting bacterial membrane transporters, known as efflux pumps (EPs), which are involved in the extrusion of noxious compounds, including antimicrobial agents [2]. EPIs have the potential to reverse resistance to several antibacterials [3,4], reduce biofilm formation associated with overexpressed EPs [4], and suppress the evolution of resistance [5]. Particularly, EPIs can be used to optimize the process of prevention of antibacterial efflux, thus boosting the efficacy of drugs acting on intracellular bacterial targets.

The need to address antimicrobial resistance mediated by NorA, one of the most studied EPs in *Staphylococcus aureus* [5,6,7], led to the identification of potent EPIs by exploitation of in silico drug repositioning approaches and medicinal chemistry campaigns [8,9,10]. We have been working for years on the design and synthesis of novel *S. aureus* NorA EPIs by comparing their biological activity on strains overexpressing *norA* or *norA*-deleted, resulting in the identification of several molecules able to fully restore the susceptibility to fluoroquinolone ciprofloxacin (CIP) of resistant *S. aureus* strains overexpressing NorA EP [8].

Knowledge of the efficacy of these compounds against bacterial species other than *S. aureus* or in combination with antimicrobial classes different from fluoroquinolones is limited to recent works reporting some derivatives also acting as nontuberculous mycobacteria EPIs [11]. However, as far as we know, no studies have considered using EPIs for pathogens of concern in the veterinary sector. Among these, *Staphylococcus pseudintermedius* has been identified by the EFSA Panel on Animal Health and Welfare among the most relevant antimicrobial-resistant bacteria due to its frequent implication in clinical diseases in dogs and cats, zoonotic risk, and the high levels of resistance to clinically relevant antibiotics that worryingly limit treatment options [12]. *S. pseudintermedius* constitutes about 90% of staphylococci isolated from healthy canine carriers and is the most prevalent cause of canine bacterial infections [13]. It is responsible for a large variety of opportunistic infections in dogs, mainly skin infections and otitis externa but also infections of the cornea, urinary, respiratory and reproductive tract [12,14,15]. These are often prolonged inflammatory disorders difficult to treat due to a high rate of biofilm production [16]. *S. pseudintermedius* is also frequently isolated from feline pyoderma [13], and it has the potential to be virulent in human hosts [17,18].

It was recently demonstrated that the overexpression of EPs in *S. pseudintermedius*, mainly NorA, is implicated in antimicrobial resistance development, particularly to CIP, which is the major metabolite of the veterinary fluoroquinolone enrofloxacin in dogs [19], gentamycin (GEN), and chlorhexidine gluconate (CHX), and it contributes to a remarkable increase of biofilm production [4]. These findings make using combinations of EPI/antibiotic or EPI/biocide an attractive strategy for preserving the efficacy of veterinary first-line drugs and antibiofilm activity. However, data concerning the relationship between the chemical structure of potential EPI compounds and the biological activity of *S. pseudintermedius* are lacking, as well as structural information about the NorA protein and its molecular interaction with EPIs.

Overall, NorA function is supposed to be conserved across the staphylococcal species [20]. However, we know that the nucleotide identity of most *S. pseudintermedius* EPs with *S. aureus* is approximately 63–73% [4]. Focusing on NorA, the *S. pseudintermedius norA* gene has a 70% nucleotide identity to *S. aureus norA* [4], corresponding to a 70% structural similarity at the protein level. These results confirm that genetic variability exists, which may impact the inhibitor design [20].

In this work, we investigated the ability of some compounds previously reported by us as *S. aureus* NorA EPIs to inhibit *S. pseudintermedius* EPs to lay the foundation of medicinal chemistry campaigns aimed at identifying novel NorA *S. pseudintermedius* EPIs.

## 2. Results

### 2.1. Synergistic Activity of EPI/Antimicrobial Combinations

**Figure 1:**
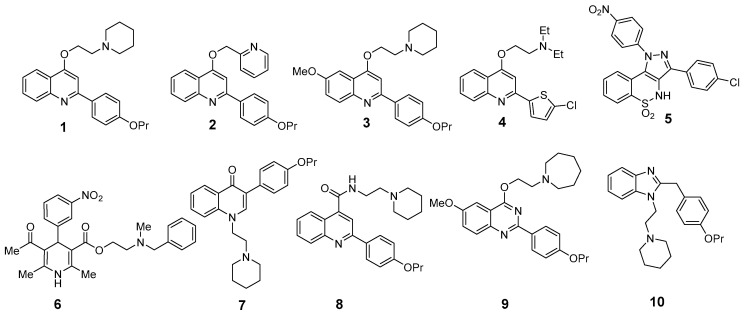
Chemical structures of the selected compounds previously reported as *S. aureus* NorA EPIs.

Ten ‘in-house’ molecules (compounds **1**–**10** depicted in Figure 1) were selected based on: (i) their own activity as *S. aureus* NorA EPIs (compound **2** was included as inactive EPI), and (ii) their structural difference in terms of central scaffold (compound **6**–nicardipine was also included since it was identified as *S. aureus* NorA EPI by a drug repurposing approach). Accordingly, seven different scaffolds can be identified within the ten selected molecules: (i) the mostly explored 2-arylquinoline present in compounds **1** [21], **2** [22], **3** [23], and **4** [24]; (ii) the pyrazolobenzothiazine in compound **5** [25]; (iii) the dihydropyridine present in nicardipine (compound **6**) [26]; (iv) the 3-phenylquinolone in compound **7** [11]; (v) the 2-phenyl-4-carboxy-quinoline in compound **8** [27]; (vi) the 2-phenyl-quinazoline in compound **9** [28]; and (vii) the benzimidazole in compound **10** [27].

To establish the concentrations of each EPI to be used in synergistic studies, we determined the Minimum Inhibitory Concentration (MIC) of ten selected compounds for *S. pseudintermedius* ATCC 49444 (wild-type) and its efflux mutant *S. pseudintermedius*_P8 (overexpressing *norA* and harboring an 11 bp deletion in the promoter region of the *norA* gene). The MICs are reported in Table 1. Overall, all compounds, when used alone, had weak antimicrobial activity against *S. pseudintermedius*, except for the pyrazobenothiazine analog **5,** which showed MIC values of 6.25 mg/L against both strains. MICs of all tested compounds exhibited the same values against both strains, suggesting that these derivatives are not *S. pseudintermedius* NorA substrates. On the other hand, being the susceptibility of *S. pseudintermedius*_P8 to these molecules affected by the increased efflux (MIC values shown in Table 1), EtBr, GEN, CIP, and CHX are considered to be substrates of *S. pseudintermedius* efflux system.

Checkerboard assays were then performed by combining EtBr, GEN, CIP, and CHX at scalar concentrations with serial dilutions of each compound used from MIC/4 to MIC/128 to prevent any synergistic effect due to an intrinsic antibacterial activity of the putative tested EPIs **1**–**10**.

When tested against *norA* overexpressing strain *S. pseudintermedius*_P8, all compounds exhibited modest to excellent synergistic activities depending on the combined antimicrobial. Compounds **1**, **3**, **5**–**8,** and **10** were synergistic with EtBr and all the antimicrobials tested (see Table 2 for modulation factor “MF” values). Of note, since all compounds were tested at serial dilutions starting from MIC/4 to MIC/128, it is essential to consider their effective concentration when comparing data (concentrations used are shown in Table 2). Overall, 2-arylquinoline derivatives **1**, **3,** and **4** exhibited a significant synergistic effect with CIP and EtBr and a weaker activity when combined with GEN and CHX. This discrepancy in synergistic activity can be imputed to a reduced efflux of these two antimicrobials. Indeed, MIC values of GEN and CHX are significantly less affected (4-fold difference between the two strains) than those of CIP and EtBr (64-fold for both). Of note, at MIC/128 (1.57 mg/L), compound **1** reduced the CIP MIC by 8-fold, thus exhibiting a very potent synergistic effect. On the contrary, the 2-arylquinoline analog **2** showed a weaker (or absent) synergistic effect in accordance with data previously obtained against *S. aureus* strains (the synergism with CIP was considered ineffective since it was not dose-dependent). Results of the pyrazolobenzothiazine derivative **5** were affected by its low MIC values, forcing us to use it at very low concentrations in checkerboard assays. However, up to MIC/8 (0.78 mg/L), compound **5** displayed a good synergistic effect (MF = 4) with GEN, CIP, and EtBr. The nicardipine drug (compound **6**) exhibited a very good profile by synergizing with GEN, CIP, and EtBr up to very low concentrations. In particular, at 1.57 mg/L (MIC/128), compound **6** still retained a MF of 4 in combination with CIP, similar to the 3-phenylquinolone analog **7** and the 2-phenyl-4-carboxy-quinoline derivative **8**. Promising results were also obtained with the quinazoline derivative **9** and the benzimidazole **10** that showed a significant synergistic effect with GEN, CIP, and EtBr up to low concentrations (6.25 and 12.5 mg/L, respectively). In addition, both of them reduced the CIP MIC 4-fold at 1.57 mg/L, thus highlighting a potent synergistic activity.

As a confirmation of the synergistic effect, the minimum fractional inhibitory concentration index (FICI) obtained for each combination EPI/antimicrobial is reported in Table 3. Data supported the potent synergism observed for most of the compounds, confirming a greater effect of the EPIs in combination with the fluoroquinolone CIP and EtBr. As expected, by also analyzing the effect of compounds in combinations with antimicrobials and EtBr against the wild-type strain (ATCC 49444), it is evident a poor or absent synergistic effect with antimicrobials (Table 3). On the other hand, a modest synergism is present when most of the compounds were combined with EtBr, which is a known nonspecific substrate of bacterial EPs, thus suggesting that most of the compounds likely possess the ability to reduce the efflux deriving by the basal expression of EPs.

### 2.2. Comparative Inhibition of EtBr Efflux

**Figure 2:**
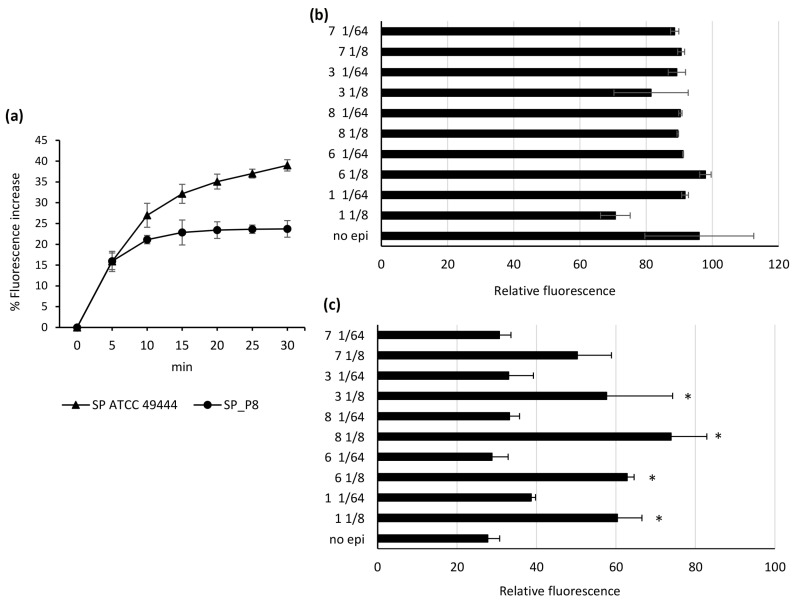
(**a**) Assessment of ethidium bromide accumulation in *Staphylococcus pseudintermedius* ATCC 49444 and *Staphylococcus pseudintermedius*_P8 in the presence of CCCP. (**b**,**c**) Relative fluorescence of ethidium bromide bound to *Staphylococcus pseudintermedius* ATCC 49444 (**b**) or P8 (**c**) DNA remaining at 30 min in the presence/absence of the compounds **1**, **3**, **6**, and **7**–**8** used at MIC/8 and MIC/64. * Statistical significance (*p* < 0.05) between no EPI treatment and treatments with compounds **1**, **3**, **6**, and **7**–**8**.

Before testing the inhibiting activity of selected compounds on *S. pseudintermedius* ATCC 49444 and *S. pseudintermedius*_P8 EPs, the capability of the bacterial strains to accumulate EtBr was tested. Predictably, EtBr accumulation was lower inside *norA*-overexpressing *S. pseudintermedius*_P8 than the parent strain (Figure 2a). Overall, the assay performed in the presence of CCCP showed a detectable accumulation for both strains.

On the strength of the data from checkerboard assays, compounds **1**, **3**, and **6**–**8** were advanced toward fluorometry-based EtBr efflux inhibition test. The EPIs were tested at MIC/8 (compounds **1** and **6**–**8**, 25 mg/L; compound **3**, 6.25 mg/L) and MIC/64 (compounds **1** and **6**–**8**, 3.13 mg/L; compound **3**, 0.8 mg/L). The results are presented in Figure 2. The use of compounds **1**, **3,** and **6**–**8** slightly influenced the EtBr efflux activity of *S. pseudintermedius* ATCC 49444 (Figure 2b). As expected, *S. pseudintermedius*_P8 showed high EtBr efflux in the absence of EPI. The residual fluorescence of EtBr in *S. pseudintermedius*_P8 was indeed 58% lower than *S. pseudintermedius* ATCC 49444, confirming the presence of highly overexpressed EPs. Compounds **3** and **6**–**8** used at MIC/64 did not alter the capability of *S. pseudintermedius*_P8 to extrude EtBr, while compound **1** at MIC/64 showed a weak inhibitory effect (Figure 2c). At the end of the test, all compounds used at MIC/8 increased EtBr fluorescence as follows: **7** 22.5% (*p* = 0.078); **3** 29.9% (*p* = 0.012); **1** 32.6% (*p* = 0.006); **6** 35.1% (*p* = 0.003); **8** 46.1% (*p* = 0.001) (Figure 2c). These findings confirm that the synergistic effect observed by checkerboard assays is due to the inhibition of drug efflux.

## 3. Discussion

The overuse and misuse of antibiotics in human and veterinary healthcare and agriculture have accelerated the emergence and spread of antimicrobial-resistant bacteria worldwide, posing a threat to the effective treatment of infections. [29,30,31]. One proposed approach to treat antibiotic-resistant bacteria is to “break” resistances by co-administering appropriate non-antibiotic drugs with failing antibiotics to re-sensitizing resistant bacteria [32]. Among the antibiotic resistance breakers, EPIs can reduce resistance by blocking bacterial EPs and thus increasing intracellular antibiotic concentration.

A wide array of compounds with EPI activity have been reported to date [32,33]. Most of them were optimized to target ESKAPE pathogens [34,35] that are considered critically important owing to their role in many human infections and the frequency of antibiotic resistance worryingly limiting treatment options [36]. Particularly, research efforts were addressed to target *S. aureus* NorA using EPIs from natural products, *de novo* synthesis, and repurposing of previously-approved drugs [8,9,10]. Pursuing the development of *S. aureus* NorA inhibitors, we have previously identified via drug repurposing approaches and medicinal chemistry campaigns a wide array of NorA EPIs characterized by different chemical scaffolds [21,22,23,25,26,28]. Most of these compounds exhibited NorA inhibition activity by restoring at very low concentrations CIP MIC against resistant *S. aureus* strains and overexpressing *norA* while not showing any effect against *S. aureus* strains not expressing *norA*.

Here, we validated the hypothesis that EPIs specifically optimized to reverse *S. aureus* NorA-mediated fluoroquinolone resistance can successfully reverse resistance to CIP, GEN, and CHX associated with the overexpression of *norA* in *S. pseudintermedius*. Our study clearly shows that most of the compounds could fully restore the susceptibility of *S. pseudintermedius* P8 to CIP, except for compound **2** (included as inactive *S. aureus* NorA EPI). This is important because enrofloxacin is largely metabolized to CIP in dogs [19]. Moreover, even if to a lesser extent, all the compounds synergized with GEN as well. On the other hand, the synergistic effect with CHX was less significant and often not showing a dose-dependent effect.

Although these results suggest that *S. aureus* NorA EPIs can also be used as *S. pseudintermedius* NorA EPIs, structure-activity relationship (SAR) information acquired over the years about *S. aureus* NorA inhibition cannot be completely transferred for designing novel *S. pseudintermedius* NorA EPIs. Indeed, the introduction of a methoxy group at the C-6 position of the quinoline core (compound **3**), as well as the replacement of the 2-phenyl ring with a chloro-thiophene moiety (compound **4**), led to an increase of the *S. aureus* NorA EPI activity with respect to “naked” 2-phenylquinoline derivatives such as compound **1**. Herein, instead, we observed that the activity of compound **1** was comparable to or slightly better than compounds **3** and **4**, thus suggesting that SAR information should be revised. Similarly, replacing the quinoline core with a quinazoline scaffold (compound **9**) led to an improvement in terms of *S. aureus* NorA inhibition activity that was not confirmed for *S. pseudintermedius* NorA EP. On the other hand, 3-phenylquinolone derivative **7**, which exhibited a weaker *S. aureus* NorA EPI activity than 2-phenylquinoline derivatives (such as compounds **1**, **3,** and **4**), retained a promising *S. pseudintermedius* NorA inhibition. Interestingly, compound **7** is also endowed with good EPI activity against nontuberculous mycobacteria resistant to clarithromycin and overexpressing EPs MAV_1406 and MAV_1695 [11]. Nicardipine (compound **6**), the carboxy-quinoline derivative **8,** and the benzimidazole analog **10** showed a comparable inhibition of both NorA EPs. The pyrazolobenzothiazine analog **5** was instead affected by low MIC values that compromised its use as EPI.

The results from the fluorometric tests provided further evidence for the capability of compounds **1**, **3**, and **6**–**8** to inhibit *S. pseudintermedius* EPs, validating their inhibition as the main mechanism involved in the synergistic effect with antimicrobials.

By combining data from checkerboard assays and EtBr efflux inhibition experiments, compounds **1**, **6,** and **8** could be considered the best NorA EPIs able to strongly synergize with CIP and EtBr against *S. pseudintermedius* P8, also showing a modest synergism with GEN and CHX. In addition, considering that their EPI activity was significantly dependent on the overexpression of *norA* EP (poor synergistic effect and no EtBr efflux inhibition were observed against the wild-type *S. pseudintermedius* strain), we are confident that these three derivatives act by inhibiting NorA efflux mechanisms. Based on the previously reported cytotoxicity evaluation of these compounds [26,27], it is interesting to note that all of them showed synergistic activity with the fluoroquinolone CIP and with the aminoglycoside GEN at concentrations significantly lower than their CC_50_ values towards human cells. Special attention should be given to nicardipine (compound **6**), which is a non-antibiotic approved drug for the treatment of high blood pressure and angina. At 3.13 mg/L, nicardipine was able to reduce CIP MIC by 8-fold (from 8 to 1 mg/L) against *S. pseudintermedius* P8 while showing a CC_50_ of 188.75 and 68.73 mg/L towards HepG2 and A549 cell lines, respectively [26]. Although further studies should be performed, nicardipine (compound **6**) could represent a promising candidate for pre-clinical studies in *in vivo* models. In parallel, results obtained for the 2-phenylquinoline derivative **1** and the 4-carboxyquinoline analog **8** give us great hope for future medicinal chemistry efforts aimed at improving *S. pseudintermedius* NorA EPI activity, safety, and pharmacokinetic profile.

Concerns have been raised questioning the therapeutic usefulness of EPIs at the community level against bacteria harboring mutations in antibiotic targets, such as gyrase-coding genes for fluoroquinolone resistance [37,38]. However, it was recently demonstrated that overexpressed EPs (specifically NorA in *S. aureus*) could create a high-resistance-evolvability bacterial niche by promoting the accumulation of antibiotic-resistance mutations or increasing the fitness benefit provided by resistance mutations [5,39,40]. Moreover, high efflux appears to be linked to the downregulation of DNA repair and mutagenesis [39]. From this perspective, EPIs might be used to prevent resistance evolution and preserve the efficacy of existing antibiotics. To further our research, future work should concentrate on using these EPI derivatives to inhibit the efflux-mediated evolvability of staphylococcal species.

## 4. Materials and Methods

### 4.1. Bacterial Strains and Growth Conditions

The strains used in this study were *S. pseudintermedius* ATCC 49444 and its derivative *S. pseudintermedius*_P8. This was obtained by adapting *S. pseudintermedius* ATCC 49444 to increasing concentrations of EtBr, a known substrate of bacterial efflux system [3,41]. More details on the growth conditions of *S. pseudintermedius*_P8 are given in our previous paper [4]. *S. pseudintermedius*_P8 overexpresses *norA* and harbors an 11 bp deletion in the *norA* promoter region. *S. pseudintermedius*_P8 shows higher MICs of EtBr, CIP, GEN, and CHX than the parent strain, as reported in Table 1.

### 4.2. Efflux Pump Inhibitors

Compounds tested as EPIs have been selected within an ‘in-house’ library of previously published *S. aureus* NorA EPIs. Seven different scaffolds identified within ten selected molecules were investigated: 2-arylquinoline present in compounds **1**, **2**, **3**, and **4**, the pyrazolobenzothiazine in compound **5**, the dihydropyridine present in compound **6**, the 3-phenylquinolone in compound **7**, the 2-phenyl-4-carboxy-quinoline in compound **8**, the 2-phenyl-quinazoline in compound **9**, and the benzimidazole in compound **10** [11,21,22,23,24,25,26,27,28].

### 4.3. MIC Determination

The lowest concentration of compounds **1**–**10** that inhibits visible bacterial growth (MIC) was determined in triplicate by broth microdilution according to CLSI recommendations [42]. A total of 96-well plates were inoculated with 100 µL of 2-fold serial dilutions of each compound in cation-adjusted Mueller-Hinton broth (CAMHB) to test a dose range of 100–3.125 mg/L. *S. pseudintermedius* ATCC 49444 and P8 colonies were resuspended in a sterile medium, and the suspension turbidity was measured spectrophotometrically at OD_600_. The bacterial suspensions were then adjusted in CAMHB to 5 × 10^5^ CFU/mL. Plates were inoculated with 100 µL of bacterial suspensions and incubated at 37 °C for 20 h.

### 4.4. Sinergy Studies

The synergistic activity of compounds **1**–**10** combined with EtBr, CIP, GEN, and CHX was evaluated on *S. pseudintermedius* ATCC 49444 and *S. pseudintermedius* P8 using two-dimensional checkerboard assays using 96-well microtiter plates according to the standards [43]. Each EPI was tested in a concentration range between MIC/4 and MIC/128. For *S. pseudintermedius* P8, the antibiotic dilutions tested ranged from the MIC to -1 doubling dilution (1 log_2_) below the MIC value for the original strain. For *S. pseudintermedius* ATCC 49444, antibiotics were tested from the MIC to MIC/8. Inoculum preparation was performed in CAMHB by colony suspension from 24 h cultures on Cation-adjusted Mueller Hinton agar. One hundred microliters of the bacterial suspension were inoculated in each well at a final concentration of 5 × 10^4^ CFUs. The plates were incubated aerobically at 37 °C for 20 h. After reading well optical turbidity, the FICI was calculated for synergy interpretation as follows:

$$\frac{MIC_{drug\;}\;combination}{MIC_{drug\;}alone}+\frac{MIC_{EPI}\;combination}{MIC_{EPI}\;alone}$$

The combination was considered synergistic when the FICI was ≤0.5. MF, Modulation Factor, represents the n-fold reduction of the MIC of the corresponding antimicrobial when combined with the EPI.

### 4.5. Fluorometric Tests

Our experimental setup is based on the one proposed by Kaatz [44]. *S. pseudintermedius* ATCC 49444 and P8 were grown overnight at 37 °C in static conditions in 10 mL of TSB without EtBr or supplemented with EtBr at 16 mg/L, respectively.

Preliminarily, we evaluated the capability of the strains to accumulate EtBr. Bacteria were pelleted by centrifugation at 8000 rpm for 10 min and washed twice with sterile PBS. The turbidity of the suspensions was adjusted to 0.6 OD_600_ in PBS. Bacterial cells were loaded with EtBr at 10 mg/L using 20 mg/L of CCCP. The fluorescence of DNA-bound EtBr was measured at 37 °C at 60 s intervals for 30 min using a TECAN Infinite 200 PRO reader at excitation/emission 530/600 nm.

To assay the inhibitory effect of selected compounds on EtBr efflux, *S. pseudintermedius* ATCC 49444 and P8 were loaded with EtBr at 10 mg/L using 20 mg/L of CCCP, as described. After 20 min of incubation at room temperature in the dark, the bacterial suspensions were pelleted, and cells were resuspended in sterile PBS at 0.6 OD_600_. One hundred microliters of each suspension were added to wells in a white microtiter plate containing, in duplicate: (i) 100 μL of PBS with glucose (final concentration 0.4%) and without EPIs (conditions of efflux); (ii) 100 μL of compound **1** at a final concentration of 25 mg/L and (iii) 3.13 mg/L in the presence of 0.4% glucose; (iv) 100 μL of compound **6** at a final concentration of 25 mg/L and (v) 3.13 mg/L in the presence of 0.4% glucose; (vi) 100 μL of compound **8** at a final concentration of 25 mg/L and (vii) 3.13 mg/L in the presence of 0.4% glucose; (viii) 100 μL of compound **3** at a final concentration of 6.25 mg/L and (ix) 0.8 mg/L in the presence of 0.4% glucose; (x) 100 μL of compound **7** at a final concentration of 25 mg/L and (xi) 3.13 mg/L in the presence of 0.4% glucose. The plate was immediately read by a TECAN Infinite 200 PRO reader at excitation/emission 530/600 nm. The fluorescence of DNA-bound EtBr was measured at 37 °C at 60 s intervals for 30 min. The assay was repeated two times. Relative fluorescence remaining at each time point was normalized against the value measured at time 0 (T_0_), as follows:

$$\frac{\left(100\times FI_{t}\right)}{FI_{t0}}$$

where *FI_t_* is the fluorescence intensity at different time points, and *FI_t_*_0_ is the fluorescence intensity at T0.

### 4.6. Statistical Analyses

Statistical analyses were performed using SPSS (version 17.0). The Mann–Whitney U test was applied to compare the biological replicates between treatments in fluorescence assays. A *p*-value < 0.05 was assumed as significant.

## 5. Conclusions

To summarize, this work provided valuable data for the medicinal chemistry optimization of EPIs for *S. pseudintermedius*. It has led us to conclude that (1) a small set of ‘in-house’ compounds optimized to reverse CIP resistance of *S. aureus* overexpressing *norA* have EPI activity against *S. pseudintermedius;* (2) all compounds synergized with CIP and GEN; (3) the synergistic effect resulted dependent on overexpressed *norA* as observed by fluorometric assays with EtBr; (4) compounds **1**, **6,** and **8** emerged as able to strongly inhibit the activity of *S. pseudintermedius* NorA EP and completely restore the activity of CIP against the resistant strain *S. pseudintermedius*_P8.

These data pave the way for further studies on antimicrobial-resistant staphylococcal species other than *S. aureus*, which have highly virulent features for both humans and companion animals.

## Figures and Tables

**Table 1:** MIC of ethidium bromide (EtBr), ciprofloxacin (CIP), gentamycin (GEN), chlorhexidine digluconate (CHX) [4], and selected EPIs for *S. pseudintermedius* ATCC 49444 and its laboratory derivative *S. pseudintermedius*_P8.

MIC (mg/L)		
Compound	SP ATCC 49444	SP_P8
**1**	>100	>100
**2**	>100	>100
**3**	50	50
**4**	25	25
**5**	6.25	6.25
**6**	>100	>100
**7**	>100	>100
**8**	>100	>100
**9**	>100	>100
**10**	>100	>100
EtBr	0.5	32
CIP	≤0.125	8
GEN	0.25	1
CHX	1	4

**Table 2:** MICs of gentamycin (GEN), chlorhexidine digluconate (CHX), ciprofloxacin (CIP), and ethidium bromide (EtBr) in the presence of compounds **1**–**10** used at scalar concentrations (ranging from 1/4 to 1/128 MIC) against *norA*-overexpressing *S. pseudintermedius*_P8.

	MIC in mg/L (MF) *								
EPI (mg/L)	GEN	CHX	CIP	EtBr	EPI	GEN	CHX	CIP	EtBr
No EPI	1	4	8	32	No EPI	1	4	8	32
1 _1/4_ (50)	**0.125 (8)**	**0.5 (8)**	**0.125 (64)**	**0.5 (64)**	2 _1/4_ (50)	**0.125 (8)**	4 (-)	**2 (4)**	16 (2)
1 _1/8_ (25)	**0.125 (8)**	**0.5 (8)**	**0.125 (64)**	**0.5 (64)**	2 _1/8_ (25)	**0.25 (4)**	4 (-)	**2 (4)**	16 (2)
1 _1/16_ (12.5)	**0.25 (4)**	**1 (4)**	**0.25 (32)**	**0.5 (64)**	2 _1/16_ (12.5)	**0.25 (4)**	4 (-)	**2 (4)**	16 (2)
1 _1/32_ (6.25)	**0.25 (4)**	2 (2)	**1 (8)**	**2 (16)**	2 _1/32_ (6.25)	**0.25 (4)**	4 (-)	**2 (4)**	16 (2)
1 _1/64_ (3.13)	0.5 (2)	2 (2)	**1 (8)**	**4 (8)**	2 _1/64_ (3.13)	0.5 (2)	4 (-)	**2 (4)**	16 (2)
1 _1/128_ (1.57)	0.5 (2)	2 (2)	**1 (8)**	**8 (4)**	2 _1/128_ (1.57)	0.5 (2)	4 (-)	**2 (4)**	32 (-)
3 _1/4_ (12.5)	**0.25 (4)**	**0.5 (8)**	**0.25 (32)**	**0.5 (64)**	4 _1/4_ (6.25)	**0.25 (4)**	2 (2)	**0.25 (32)**	**0.5 (64)**
3 _1/8_ (6.25)	**0.25 (4)**	**1 (4)**	**0.5 (16)**	**1 (32)**	4 _1/8_ (3.13)	**0.25 (4)**	2 (2)	**0.5 (16)**	**2 (16)**
3 _1/16_ (3.13)	0.5 (2)	2 (2)	**1 (8)**	**4 (8)**	4 _1/16_ (1.57)	0.5 (2)	2 (2)	**2 (4)**	**8 (4)**
3 _1/32_ (1.57)	0.5 (2)	2 (2)	**2 (4)**	**8 (4)**	4 _1/32_ (0.78)	0.5 (2)	2 (2)	4 (2)	16 (2)
3 _1/64_ (0.78)	0.5 (2)	4 (-)	**2 (4)**	32 (-)	4 _1/64_ (0.39)	0.5 (2)	2 (2)	4 (2)	16 (2)
3 _1/128_ (0.39)	0.5 (2)	4 (-)	4 (2)	32 (-)	4 _1/128_ (0.20)	1 (-)	4 (-)	8 (-)	32 (-)
5 _1/4_ (1.57)	**0.25 (4)**	**1 (4)**	**0.5 (16)**	**4 (8)**	6 _1/4_ (50)	**0.125 (8)**	**1 (4)**	**0.25 (32)**	**0.5 (64)**
5 _1/8_ (0.78)	**0.25 (4)**	2 (2)	**2 (4)**	**8 (4)**	6 _1/8_ (25)	**0.125 (8)**	**1 (4)**	**0.25 (32)**	**0.5 (64)**
5 _1/16_ (0.39)	0.5 (2)	2 (2)	**2 (4)**	16 (2)	6 _1/16_ (12.5)	**0.25 (4)**	2 (2)	**0.5 (16)**	**1 (32)**
5 _1/32_ (0.20)	0.5 (2)	2 (2)	**2 (4)**	16 (2)	6 _1/32_ (6.25)	0.5 (2)	2 (2)	**1 (8)**	**8 (4)**
5 _1/64_ (0.10)	0.5 (2)	2 (2)	**2 (4)**	16 (2)	6 _1/64_ (3.13)	0.5 (2)	2 (2)	**1 (8)**	16 (2)
5 _1/128_ (0.05)	1 (-)	4 (-)	4 (2)	32 (-)	6 _1/128_ (1.57)	1 (-)	4 (-)	2 (4)	32 (-)
7 _1/4_ (50)	**0.25 (4)**	**1 (4)**	**0.25 (32)**	**0.5 (64)**	8 _1/4_ (50)	**0.25 (4)**	**0.5 (8)**	**0.25 (32)**	**0.5 (64)**
7 _1/8_ (25)	**0.25 (4)**	2 (2)	**0.25 (32)**	**2 (16)**	8 _1/8_ (25)	**0.25 (4)**	**0.5 (8)**	**0.25 (32)**	**0.5 (64)**
7 _1/16_ (12.5)	**0.25 (4)**	2 (2)	**0.5 (16)**	**8 (4)**	8 _1/16_ (12.5)	**0.25 (4)**	2 (2)	**0.25 (32)**	**2 (16)**
7 _1/32_ (6.25)	0.5 (2)	2 (2)	**1 (8)**	16 (2)	8 _1/32_ (6.25)	**0.25 (4)**	2 (2)	**0.5 (16)**	**8 (4)**
7 _1/64_ (3.13)	0.5 (2)	2 (2)	**1 (8)**	16 (2)	8 _1/64_ (3.13)	0.5 (2)	2 (2)	**1 (8)**	16 (2)
7 _1/128_ (1.57)	1 (-)	4 (-)	**2 (4)**	32 (-)	8 _1/128_ (1.57)	0.5 (2)	4 (-)	**2 (4)**	32 (-)
9 _1/4_ (50)	**0.25 (4)**	4 (-)	**0.5 (16)**	**2 (16)**	10 _1/4_ (50)	**0.25 (4)**	**1 (4)**	**0.5 (16)**	**8 (4)**
9 _1/8_ (25)	**0.25 (4)**	4 (-)	**1 (8)**	**2 (16)**	10 _1/8_ (25)	**0.25 (4)**	**1 (4)**	**0.5 (16)**	**8 (4)**
9 _1/16_ (12.5)	**0.25 (4)**	4 (-)	**1 (8)**	**2 (16)**	10 _1/16_ (12.5)	**0.25 (4)**	2 (2)	**0.5 (16)**	**8 (4)**
9 _1/32_ (6.25)	**0.25 (4)**	4 (-)	**1 (8)**	**4 (8)**	10 _1/32_ (6.25)	0.5 (2)	2 (2)	**1 (8)**	16 (2)
9 _1/64_ (3.13)	0.5 (2)	4 (-)	**2 (4)**	**8 (4)**	10 _1/64_ (3.13)	0.5 (2)	2 (2)	**2 (4)**	16 (2)
9 _1/128_ (1.57)	0.5 (2)	4 (-)	**2 (4)**	16 (2)	10 _1/128_ (1.57)	1 (-)	4 (-)	**2 (4)**	32 (-)

* MF: Modulation Factor that represents the n-fold reduction of the MIC of the corresponding antimicrobial when combined with the EPI. Hyphen (-) indicates no reduction of the MIC. Bold numbers indicate Modulation Factors (MF) ≥ 4.

**Table 3:** The lowest Fractional Inhibitory Concentration Index (FICI) obtained by combining compounds **1**–**10** with ethidium bromide (EtBr), ciprofloxacin (CIP), gentamycin (GEN), and chlorhexidine digluconate (CHX) for *S. pseudintermedius* ATCC 49444 and *S. pseudintermedius*_P8. Bold numbers indicate synergistic combinations.

	MIC (mg/L)							
	GEN		CHX		CIP		EtBr	
EPI	SP 49444	SP P8	SP 49444	SP P8	SP 49444	SP P8	SP 49444	SP P8
1	**0.50**	**0.25**	0.56	**0.25**	**0.50**	**0.13**	**0.16**	**0.08**
2	2.00	**0.28**	2.00	1.00	2.00	**0.26**	2.00	0.51
3	2.00	**0.38**	0.75	**0.38**	2.00	**0.16**	**0.50**	**0.16**
4	0.56	**0.38**	2.00	0.53	2.00	**0.19**	**0.25**	**0.19**
5	0.56	**0.38**	2.00	**0.50**	2.00	**0.28**	2.00	**0.38**
6	**0.50**	**0.31**	0.56	**0.38**	2.00	**0.13**	**0.19**	**0.09**
7	**0.50**	**0.31**	0.56	**0.50**	2.00	**0.13**	**0.50**	**0.19**
8	2.00	**0.28**	**0.50**	**0.25**	2.00	**0.09**	**0.25**	**0.13**
9	2.00	**0.28**	0.56	1.00	2.00	**0.16**	0.56	**0.09**
10	**0.38**	**0.31**	0.56	**0.38**	2.00	**0.13**	**0.25**	**0.31**

## Data Availability

The data presented in this study are available upon request from the corresponding author.
